# Supplementary material for: Piezo activity levels need to be tightly regulated to maintain normal morphology and function in pericardial nephrocytes
Source: Sci Rep. 2024 Nov 16;14:28254. doi: 10.1038/s41598-024-79352-9 (PMC11568303; doi:10.1038/s41598-024-79352-9)
Supplement: Supplementary file 1 — Supplementary Material 1 [file 41598_2024_79352_MOESM1_ESM.pdf]

Supplementary data

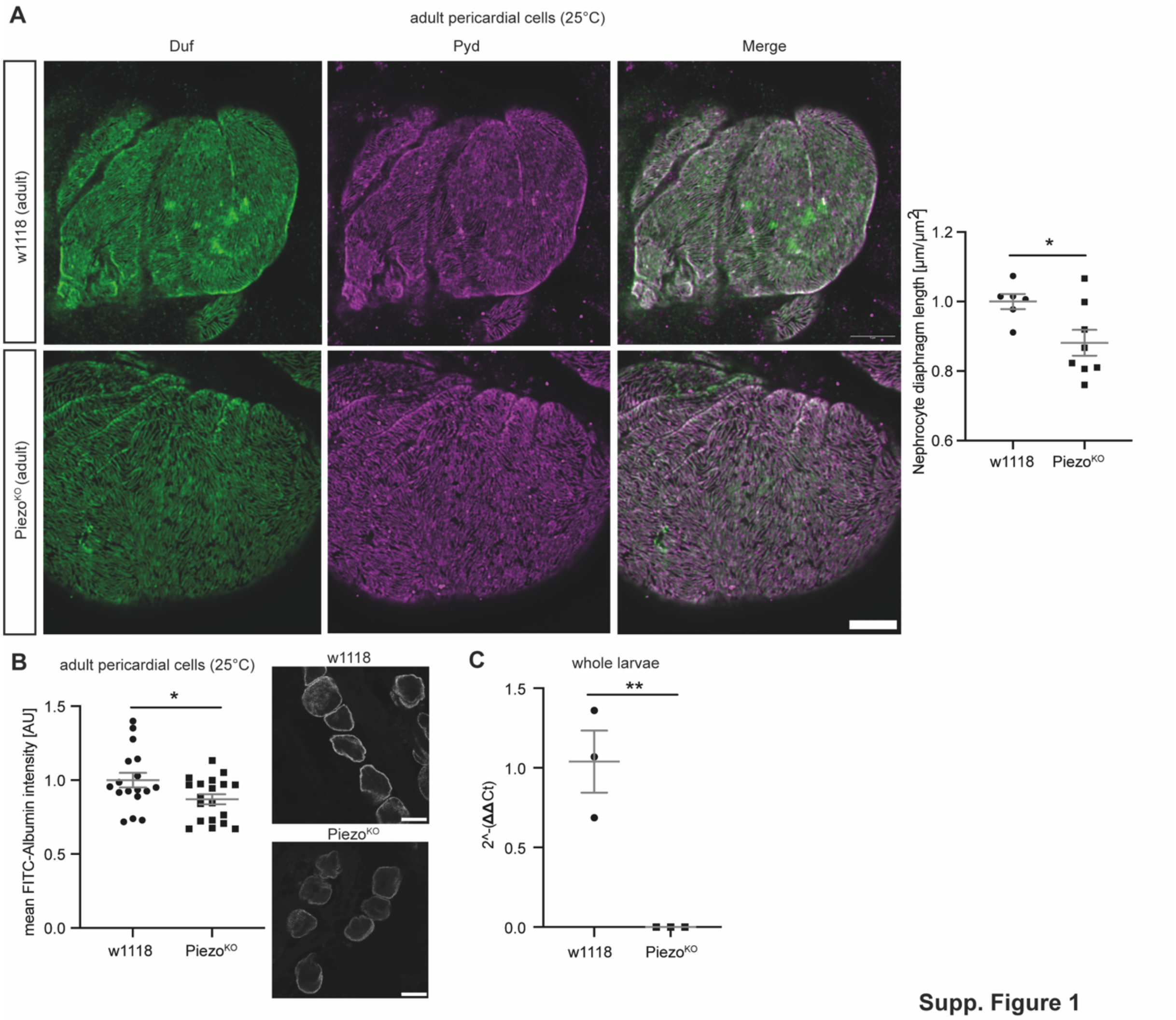

Supp. Figure 1: Piezo knockout flies present with a phenotype in adult pericardial cells. A Visualization of the nephrocyte diaphragm with Duf and Pyd antibodies reveals a significant decrease in nephrocyte diaphragm length in adult pericardial cells isolated from Piezo whole body knockout flies (Piezo^{KO}). As controls w1118 flies have been used, as the Piezo^{KO} is backcrossed into this strain. Student's t-test: *: $p<0.05$. Scale bar = 5µm. **B** FITC-Albumin uptake assays reveal a significant decrease of FITC-Albumin at the cell cortex in Piezo^{KO} pericardial cells. Scale bar = 25µm. Student's t-test: *: $p<0.05$. **C** Q-PCR of whole Piezo^{KO} larvae show a highly significant decrease of Piezo expression, confirming the KO efficiency. Student's t-test: **: $p<0.01$.

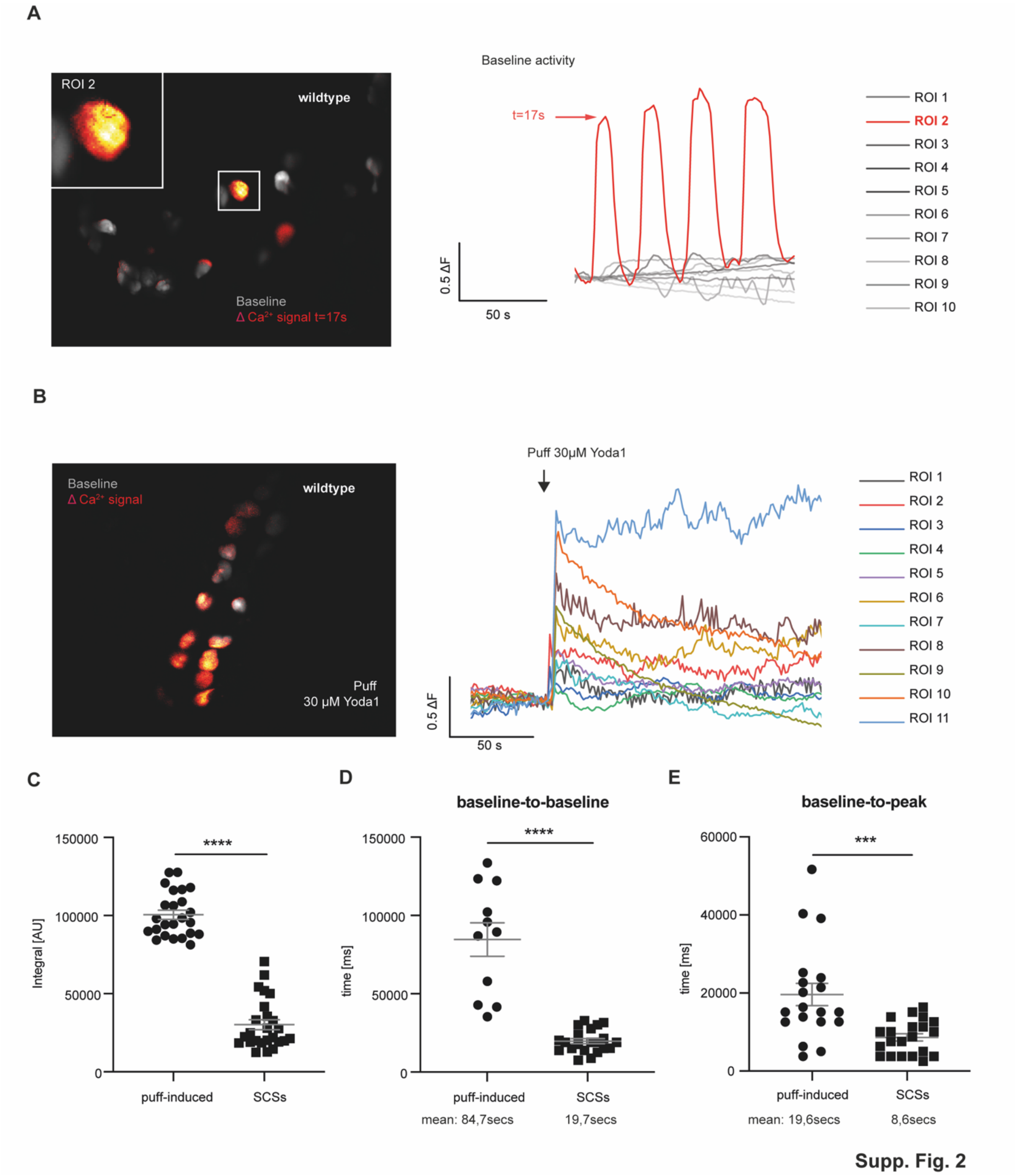

Supp. Figure 2: Calcium imaging in pericardial nephrocytes. A The image (left) shows spontaneous Ca^{++} activity (red) in GCamP6 expressing nephrocytes. Each pericardial cell was marked as an ROI and changes in calcium levels over the course of an experiment are depicted in corresponding traces (right). ROI2 shows the occurrence of spontaneous calcium signals. **B** The image (left) shows synchronous puff-induced calcium signals (red) in the majority of GCamP6-expressing nephrocytes and the corresponding traces of each ROI (right). **C** Quantification of the size of the integral of puff-induced (mean of all ROIs in one fly represent a datapoint, 23 flies in total) and spontaneous calcium signals (every SCSs in 11ROIs in 9 flies).; Student's t-test: ****: $p < 0.0001$. **D** Quantification of baseline-to-baseline duration of puff-induced (mean of 11 flies, in which signals went back to baseline) and spontaneous calcium signals (every SCSs in 11ROIs in 9 flies). Student's t-test: ****: $p < 0.0001$. **E** Quantification of baseline-to-peak duration in puff-induced (mean of 19 flies) and spontaneous calcium signals (every SCSs in 11ROIs in 9 flies). Student's t-test: *** $p < 0.001$.

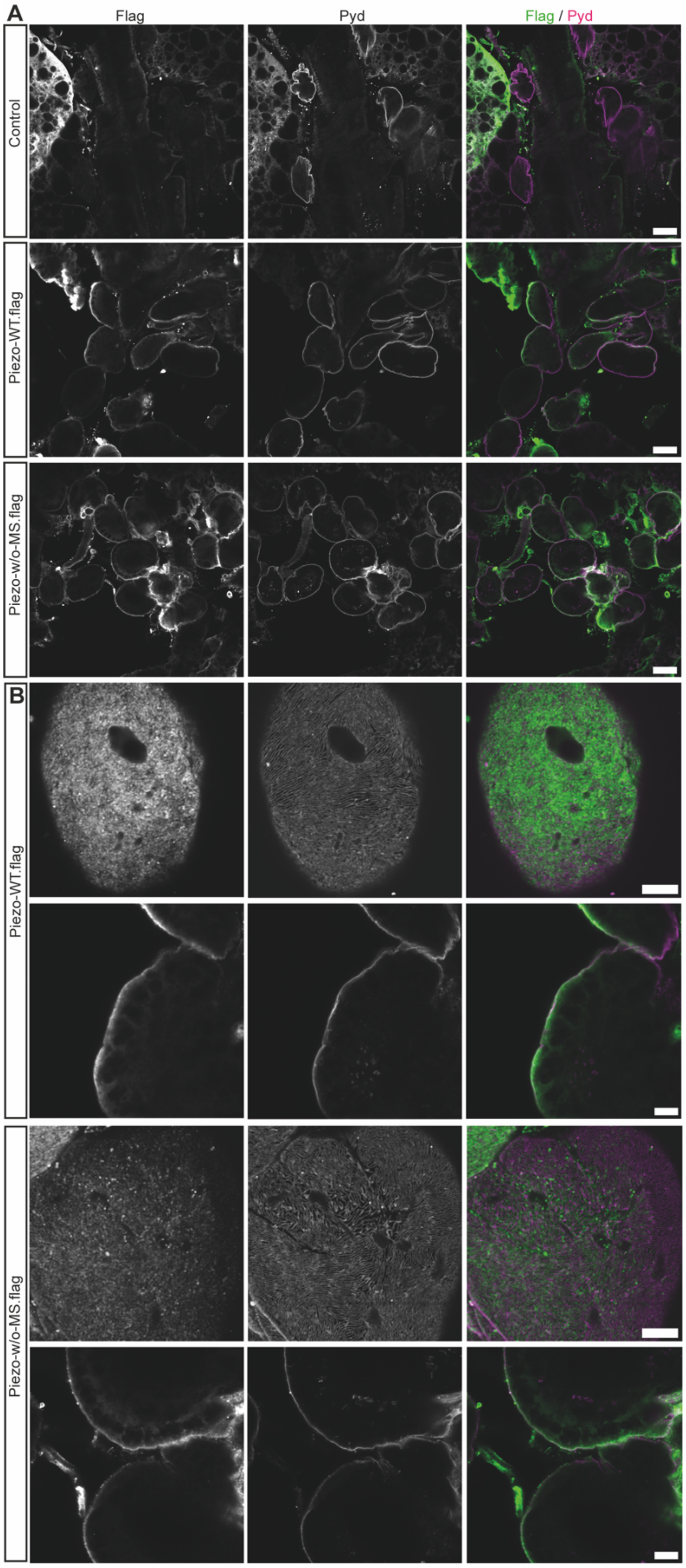

Supp. Fig. 3

Supp. Figure 3: Overexpression of Piezo wildtype and a Piezo mutant lacking the mechanosensitive domain. A Immunofluoresence of adult pericardial nephrocytes expressing Piezo wildtype or the mutant Piezo at 25°C visualizing the nephrocyte diaphragm with a Pyd antibody and the flag tag to visualize Piezo. Scale bar = 25µm (upper three rows). Control: w; *sns*-Gal4/+;UAS-*dicer2*/+. Piezo WT: w; *sns*-Gal4/+;UAS-*dicer2*/UAS-*piezo*-flag. Piezo w/o MS: w; *sns*-Gal4/+;UAS-*dicer2*/UAS-*piezo*-2306MYC.flag. **B** Higher magnification images of pericardial cells to show the flag tag expression at the surface and the cortex of pericardial cells expressing Piezo wildtype or the mutant. Scale bar = 5µm.

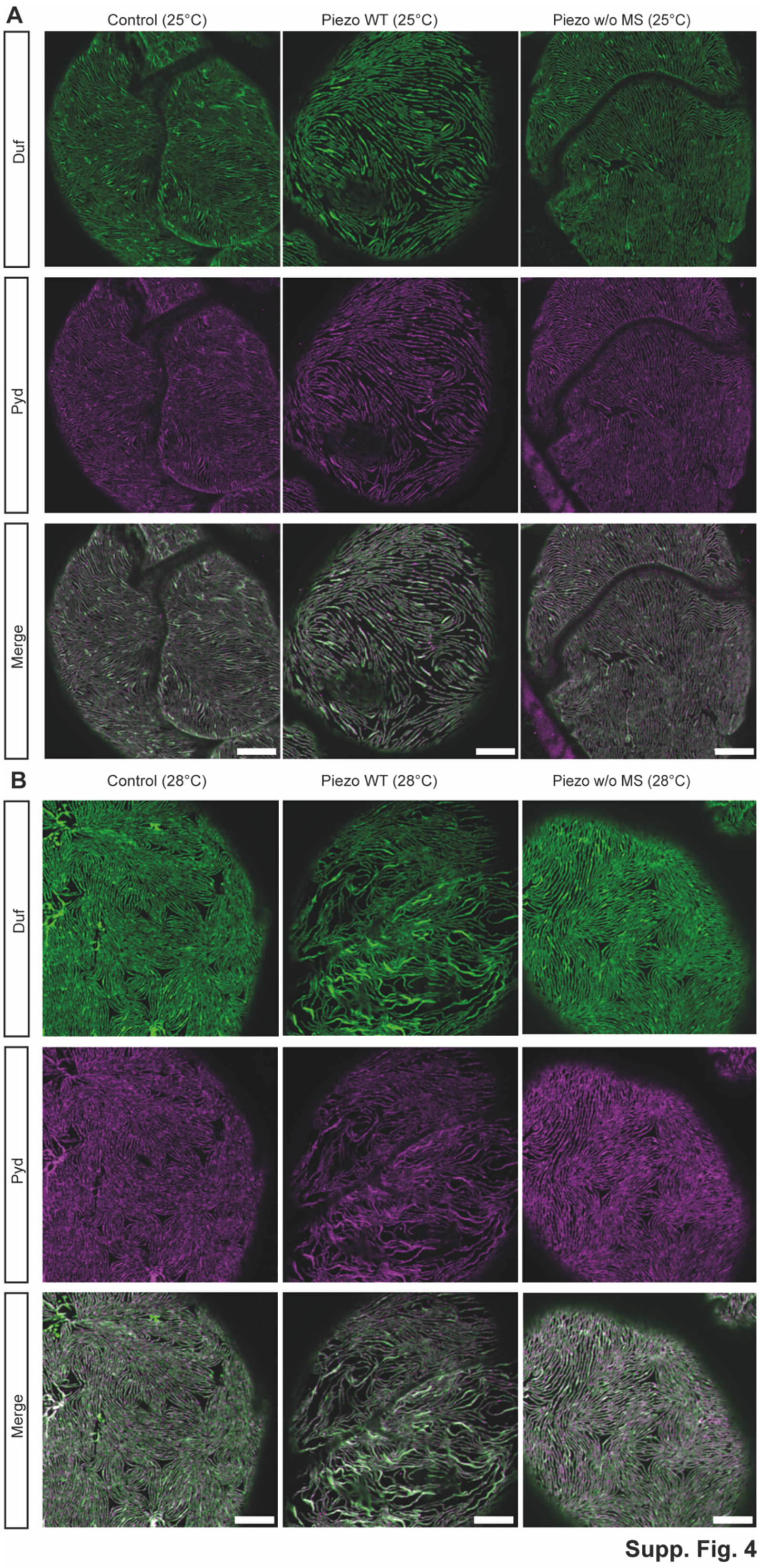

Supp. Figure 4: Overexpression of Piezo causes a nephrocyte phenotype, which is mediated by the mechanosensitive channel domain. A,B Immunofluoresence of adult pericardial nephrocytes expressing Piezo wildtype or the mutant Piezo at 25°C (medium levels) and 28°C (high levels) visualizing the nephrocyte diaphragm with Duf and Pyd antibodies. Scale bar = 5µm. Control: w; *sns*-Gal4/+;UAS-*dicer2*/+. Piezo WT: w; *sns*-Gal4/+;UAS-*dicer2*/UAS-*piezo*-flag. Piezo w/o MS: w; *sns*-Gal4/+;UAS-*dicer2*/UAS-*piezo*-2306MYC.flag.

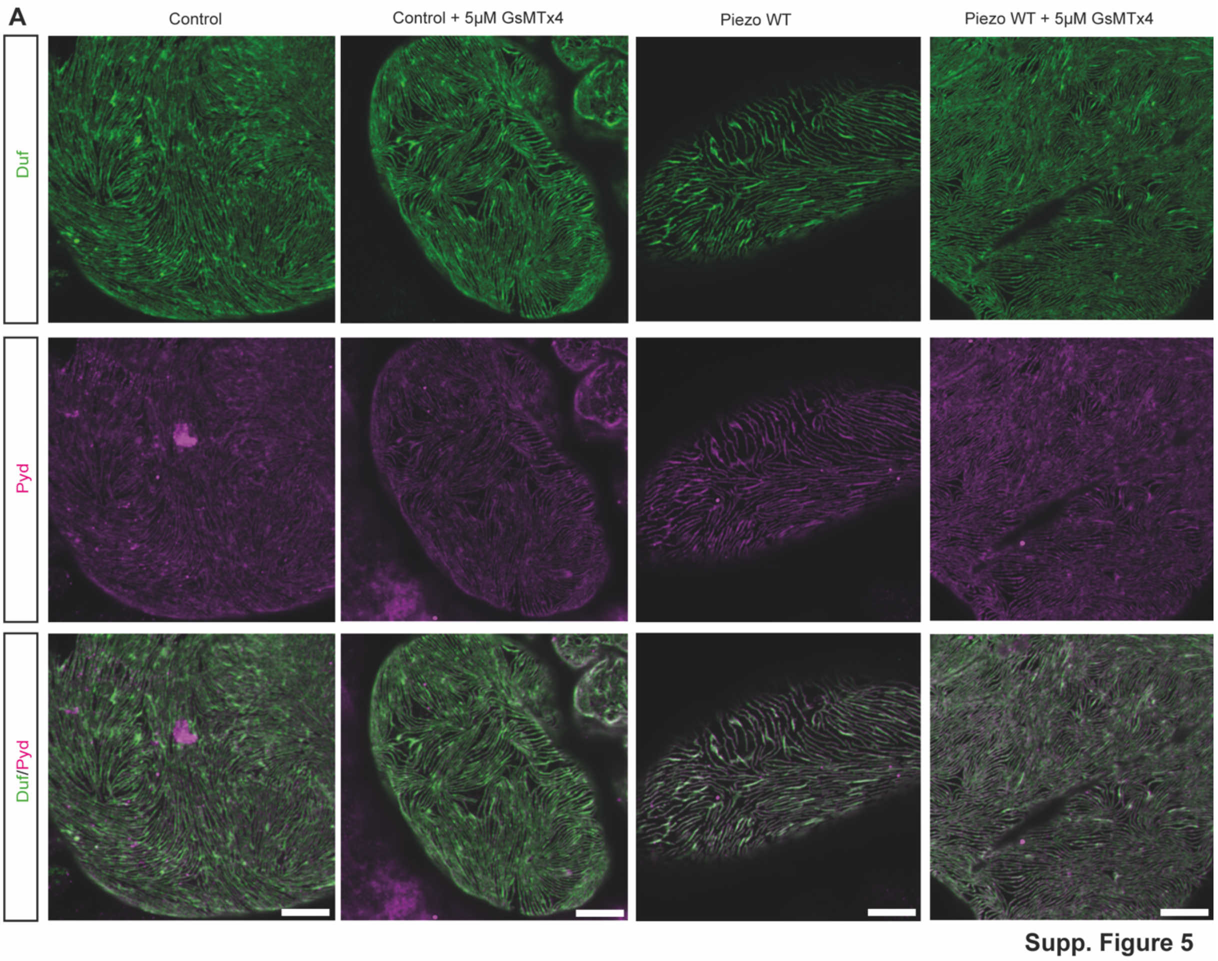

Supp. Figure 5: GsMTx4 treatment reverses the morphological phenotype observed in adult pericardial nephrocytes overexpressing Piezo wildtype. A Immunofluorescence staining with Duf and Pyd antibodies reveals a rescue of the morphological changes after incubation with 5μM GsMTx4 for 5mins in adult pericardial cells expressing Piezo wildtype. Scale bar = 5μm. Control: w; *sns*-Gal4/+;UAS-*dicer2*/+. Piezo WT: w; *sns*-Gal4/+;UAS-*dicer2*/UAS-*piezo*-flag.
